# Vacuum-powered soft actuator with oblique air chambers for easy detachment of artificial dry adhesive by coupled contraction and twisting

**DOI:** 10.1080/14686996.2023.2274818

**Published:** 2023-11-09

**Authors:** Seung Hoon Yoo, Minsu Kim, Han Jun Park, Ga In Lee, Sung Ho Lee, Moon Kyu Kwak

**Affiliations:** a Department of Mechanical Engineering, Kyungpook National University, Daegu, Republic of Korea; b Department of Mechanical Engineering, Dong-A University, Busan, Republic of Korea

**Keywords:** Soft actuator, dry adhesive, gecko, robot arm, glass transportation systems

## Abstract

A gecko foot-inspired, mushroom-shaped artificial dry adhesive exploiting intermolecular forces between microstructure and surface has drawn research attention for its strong adhesive force. However, the high pull-off strength corresponding to the adhesive force matters when detaching fragile substrates. In this study, we report a vacuum-powered soft actuator having oblique air chambers and a dry adhesive. The soft actuator performs coupled contraction and twisting by applying negative pneumatic pressure inward and exhibits not only high pull-off strength but also easy detachment. This effective detachment can be achieved thanks to the twisting motion of the soft actuator. The detachment performances of the actuator models are assessed using a 6-degrees-of-freedom robot arm. Results show that the soft actuators exhibit remarkable pull-off strength decrement from ~20 N cm^−2^ to ~2 N cm^−2^ due to the twisting. Finally, to verify a feasible application of this study, we utilize the inherent compliance of the actuators and introduce a glass transfer system for which a glass substrate on a slope is gripped by the flexibility of the soft actuators and delivered to the destination without any fracture.

## IMPACT STATEMENT

Vacuum-powered soft actuator with oblique air chambers for easy detachment of artificial dry adhesive by coupled contraction and twisting

In this work, we report a vacuum-powered soft actuator with artificial dry adhesive. We used a vacuum for the easy detachment of the dry adhesive and verified a feasibility for the real-industrial fields. The highlights and novelty of this work are as follows:

• Vacuum-powered soft actuator with Gecko inspired dry adhesive

• High adhesion with an easy detachment by twisting motion

• Mechanical analysis of the easy detachment

• Novel glass transfer system with the 6-DOF robot arm based on the soft actuator

## GRAPHICAL ABSTRACT

**Figure:**
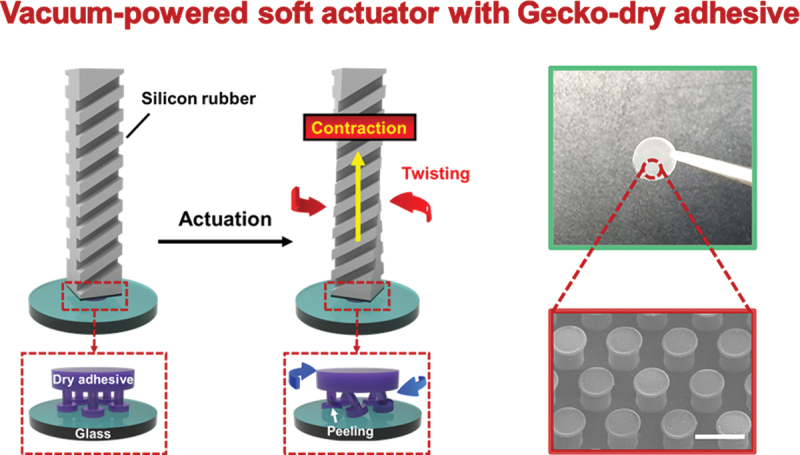

## 1. Introduction

Gecko lizards are known for their superior ability to climb vertical surfaces or even move upside down on a ceiling at speeds of up to 77 cm s^−1^. The key feature of a gecko’s astonishing adhesion lies in the micro-setae on their toes. Each micro-seta, approximately 100 μm in length and 5 μm in diameter, is formed from β-keratin, and its tip branches into spatulae consisting of stalks with flat triangular ends. When the setae are planted on a surface, intermolecular surface forces, such as the van der Waals force, are generated at the interface, which is strong enough to exceed the entire weight of a lizard [1–5]. To utilize the adhesion mechanism of a gecko’s foot, studies have proposed the use of mushroom-shaped artificial dry adhesives consisting of a hierarchical bilayer comprising micropillars and tips, which exhibit good adhesion, repeatability, and self-cleaning capability compared to nanostructures with simple spatulate tips [6–11]. Given these advantages, glass transfer systems with dry adhesive have been introduced to deliver a heavy, wide, thin glass with a robot arm or a gripper [10,12–14]. However, the high pull-off strength derived from detachment in the vertical direction can damage or even fracture a fragile glass substrate, such as a wide and thin liquid crystal display. To detach the dry adhesive with low pull-off strength, the application of shear force on the interface has been introduced [15–20]. This method decreases the pull-off strength, as it induces a peeling effect of the micropillars and reduces the real contact area of the attached glass. Inspired by this principle, a novel detachment method has been introduced, which exerts shear force on the interface by twisting the dry adhesive, thus generating a low pull-off strength that is approximately half to the detachment in the vertical direction [21].

These days, research in terms of ‘robotics’ have been actively carried out with the development of software such as artificial intelligence (AI) and machine learning (ML), and various studies related to ‘soft robots’ have also been conducted. Unlike conventional robots for routine practices with restricted environment, soft robots feature versatile shape deformation and high adaptability in various environments [22–25]. Furthermore, unlike conventional hard robots constructed with barely deformable substances, such as metal and aluminum alloy, soft robots deform themselves and exhibit a wide range of motions, such as swimming, crawling, and galloping, in response to their inherent compliance with their base materials [26–30]. Among soft robots, a soft pneumatic actuator is one of the popular actuating methods. It is generally fabricated using flexible materials, such as silicone rubber, and typically includes embedded monolithic air network with multiply connected air chambers. By applying pneumatic pressure on the network, the actuator can perform complex motions such as extension, contraction, bending, and twisting [31–37]. Utilizing these smart actuators, research have been conducted to grip and convey smooth glasses [38–40] in recent years. However, rupture of the glass owing to excessive pull-off force was not sincerely considered in the previous studies yet.

**Figure 1.**
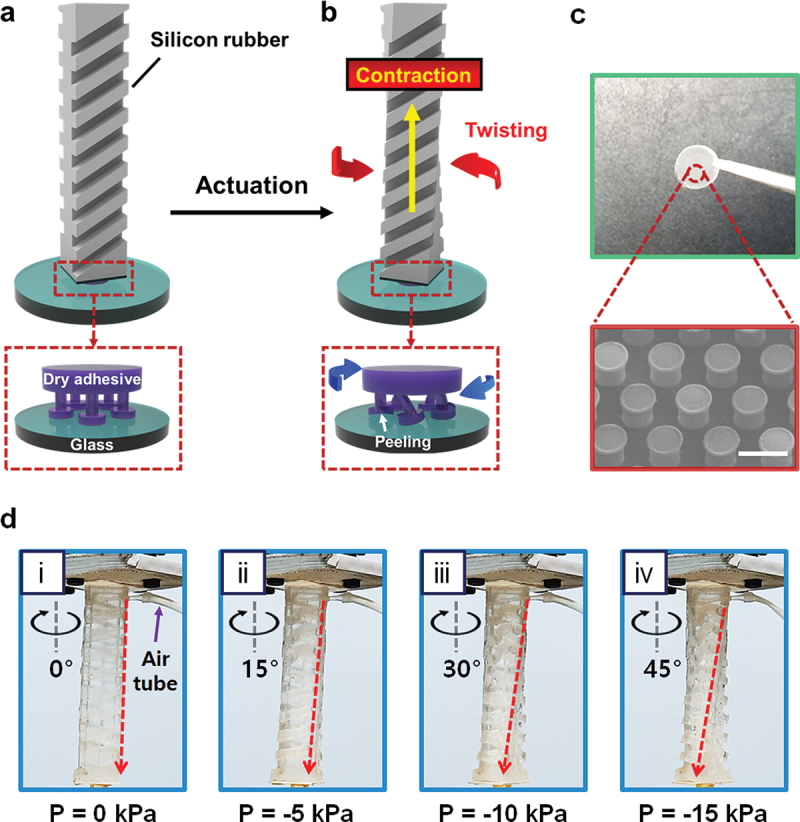
Concept of vacuum-powered soft actuator and its operation. (a) Combination of the actuator and mushroom-shaped artificial dry adhesive attached to a glass. (b) Complex motions of the actuator with the detachment of micropillars by vacuum pneumatic pressure. (c) A photograph of circular dry adhesive specimen with SEM image of micropillars. The scale bar is 20 μm. (d) Actuating of the soft actuator according to negative pressures: (i) 0 kPa, (ii) −5 kPa, (iii) −10 kPa, and (iv) −15 kPa.

In this study, we report a vacuum-powered soft actuator with the mushroom-shaped artificial dry adhesive, as shown in Figure 1. The actuator exhibits not only strong adhesion but also easy detachment with glass and contains oblique air chambers that can be deformed easily by controlling air pressure. Several models focusing on the influence of the angle of air chambers and dimension were demonstrated through a characteristic equation governing the design of the actuators, and prototypes were fabricated with the dry adhesive using soft lithography. The performances of the actuator models, such as high pull-off strength and easy detachment, were verified with a 6-degrees-of-freedom (6-DOF) robot arm. Then, the results were compared with previous soft glass grippers focusing on pull-off force and its switching. Finally, a novel glass transfer system that can attach a tilted glass by bending and safely detach after delivery was also introduced as a feasible application.

## 2. Experimental sections

### 2.1. Fabrication of the artificial dry adhesive inspired by the gecko foot

The gecko-inspired, mushroom-shaped artificial dry adhesive was fabricated through conventional soft lithography with polydimethylsiloxane (PDMS) (Figure S1) [7]. First, the elastomer base and curing agent (Sylgard 184 kit, Dow Corning Corp, U.S.A.) were mixed in a ratio of 10:1, and the PDMS mixture was degassed to remove air bubbles in the vacuum chamber. Then, the degassed PDMS mixture was poured onto a master mold with mushroom-shaped microstructures and cured in a 70°C oven for 2 h. The cured PDMS was carefully demolded from the master mold (Supplementary Figures S1(a–c)). The total thickness of the sample was controlled at ~2 mm, and each micropillar had a height of 20 μm, diameter of 10 μm, tip thickness of 1 μm, and tip diameter of 13 μm. The pillar arrays were arrayed in a hexagonal pattern 20 μm away from each pillar’s center.

### 2.2. Preparation of the vacuum-powered soft actuator

Vacuum-powered soft actuator models were fabricated by employing a simple process of soft lithography depicted in Supplementary Figure S2. The mold for the soft actuator body was produced by a digital light processing-type 3D printer with ultraviolet light-curable resin and overcured for 12 h after printing to increase the solidity of the molds (Supplementary Figures S2(a-i)). Then, the left and right parts of the mold were slightly joined together, and the degassed PDMS mixture was poured on it (Supplementary Figure S2(a-ii)). After an additional 30 minutes of deaeration, the mold was completely assembled and cured in the 70°C oven for 2 h (Supplementary Figure S2(a-iii)). Half of the soft actuator body was fabricated through this process, and it was repeated for the other. The fabricated two halves were assembled back-to-back, and the actuator body was completely fabricated (Supplementary Figures S2a(iv and v)). Finally, by firmly bonding the artificial dry adhesive with a 1.7-mm-thick thin glass (weight: 12 g) on the top of the soft actuator, the preparation of the vacuum-powered soft actuator was finished (Supplementary Figure S2(b)).

### 2.3. Experimental setup with a 6-DOF robot arm

The detachment experiments of the soft actuator models were conducted to measure the generated pull-off strength using custom-built equipment. A 6-DOF robot arm (MZ07, Nachi, Japan), actuator models, an Arduino-based pneumatic pump kit, and load cell sensors on which a glass was fixed were prepared for the tests. In all experiments, the soft actuator was assembled with the robot arm and was in contact conformally with the glass. At first, after a preload of 5 N cm^−2^ was given to the dry adhesive by the robot arm before the initiation of each implement (Supplementary Figure S3(a)), the robot arm lifted the glass with a velocity of 0.5 mm s^−1^ to apply a normal force in a vertical direction on the interface between the dry adhesive and glass (Supplementary Figure S3(b)). Second, the soft actuator was operated by applying negative pneumatic pressure by the pump kit after 1 s from the initiation to induce contraction and twisting on the interface at the same time (Supplementary Figures S3(c,d)). During the experiment, pull-off strength and applied negative air pressure were evaluated simultaneously with a sampling time interval of 100 ms. Through 10 iterations, the average value was obtained and then used in the experimental data. If the dry adhesive was successfully detached by the actuation, the measurements were repeated after increasing its diameter. Otherwise, the model was excluded from further measurements.

## 3. Results and discussion

### 3.1. Design of the soft actuator

The soft actuator in this article was based on finger-shaped structures, and the artificial dry adhesive having a strong adhesion was assembled at the end of the soft actuator. Figure 1 provides an overview of the entire soft actuator. As depicted in Figure 1(a,b), once the pneumatic force is applied to the soft actuator, a twisting motion of the soft actuator is induced, and torque is generated. The dry adhesive is composed of hierarchical microstructures resembling a ‘mushroom’ shape, and according to the literature, a twisting motion facilitates the easy detachment of dry adhesive [21]. Therefore, easy detachment of the dry adhesive can be achieved using the twisting motion. This facile mechanism would be explained later in detail. First of all, to select the base material for the soft actuator, a consideration of stiffness was needed. Due to the high adhesion of the dry adhesive, high stress must be exerted from soft pneumatic actuators to detach the artificial dry adhesive [7]. Given that PDMS features higher stiffness (Young’s modulus = ~1.8 MPa) compared to EcoFlex (Young’s modulus = ~0.1 MPa), which is a well-established silicone elastomer for soft pneumatic actuators, the PDMS was selected as the base material in this study [41].

The next part is about the twisting motion of the soft actuator, which is the key feature of this study. As Mosadegh’s work demonstrated [31], a finger-shaped actuator with an integrated pneumatic system consisting of a single main channel and air chambers exhibits bending when pneumatic pressure is internally applied. In accordance with finite element analysis and the experimental results found in the literature, the soft pneumatic actuators with oblique air chambers could exhibit coupled bending and twisting [34,42]. Furthermore, the overlapped arrays of equivalent air chambers achieve a higher twisting angle compared to the arrays located side by side [33]. From these backgrounds, each soft actuator model is designed to be overlapped by the same halves, embed a pneumatic network with oblique air chambers, and generate normal force and torque derived from coupled contraction and twisting when negative pneumatic pressure is applied. As shown in Figures 1(d), the soft actuator has successfully shown various twisting motions according to the pressure.

**Figure 2.**
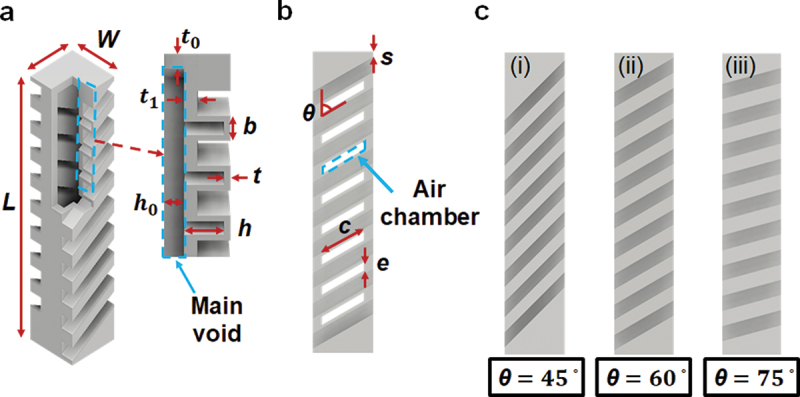
Schematic view of the design of a soft pneumatic actuator. (a) The fixed design parameters of an actuator model. (b) The variable design parameters of the actuator model. (c) The proposed models with air chamber angle (i) θ = 45°, (ii) θ = 60°, and  (iii) θ = 75°, respectively.

Figure 2 shows the whole information of the soft actuator and various design parameters in detail. In particular, Figure 2(a) presents the finger shape with a cross-sectional area of 20 mm × 20 mm and height of 100 mm. As can be seen, each actuator has a space for a main void in the middle of the structure and air chambers of oblique shape. The same halves of the actuator are stacked together to offset bending and induce twisting when pneumatic pressure is applied inward. Other fixed design parameters of the actuator models are as follows: the height of the air chamber (*h* = 6 mm), the height of the main void ($h_{O}$ = 3 mm), wall thickness between the outside part and air chamber (*t* = 1 mm), the width of air chamber (*b* = 5 mm), and wall thickness between the pneumatic network and outside part ($t_{0}$ = 3 mm and $t_{1}$ = 2 mm).

Based on the fixed parameters, the soft actuators were tuned by determining several variable parameters. Figure 2(b) shows the top side of a model that is visible on the inward edges of air chambers. The characteristic equation that governs the geography of each model is as follows:

(1) $$2s+b\left(2N+1\right)+W\;tan\left(90^{\circ }-\theta \right)=L$$

where *θ* is the angle of air chambers and *N* and *s* are the numbers of air chambers and distance s at the edge, respectively.

To investigate the effect of angle *θ*, *N* and *s* were determined after *θ*. Table 1 presents the values of *s* and *N* corresponding to the specific value of *θ* from *θ* = 15° to 75° at a fixed value of *L* = 100 mm. The designs of angle *θ* = 0° and 90° were excluded because only contraction occurred without twisting when vacuum pressure was applied [34,43]. The designs of angle values *θ* = 15° and 30° were also excluded as they contained low numbers of *N*. As a result, three models of *θ* = 45°, 60°, 75° are proposed, as shown in Figure 2(c).

**Table 1.** Information for the geography of various soft actuators.

θ (degree)	75	60	45	30	15
*N* (#)	8	8	7	5	2
*S* (mm)	4.82	1.73	2.50	5.18	0.18

### 3.2. Performance of the soft actuator

**Figure 3.**
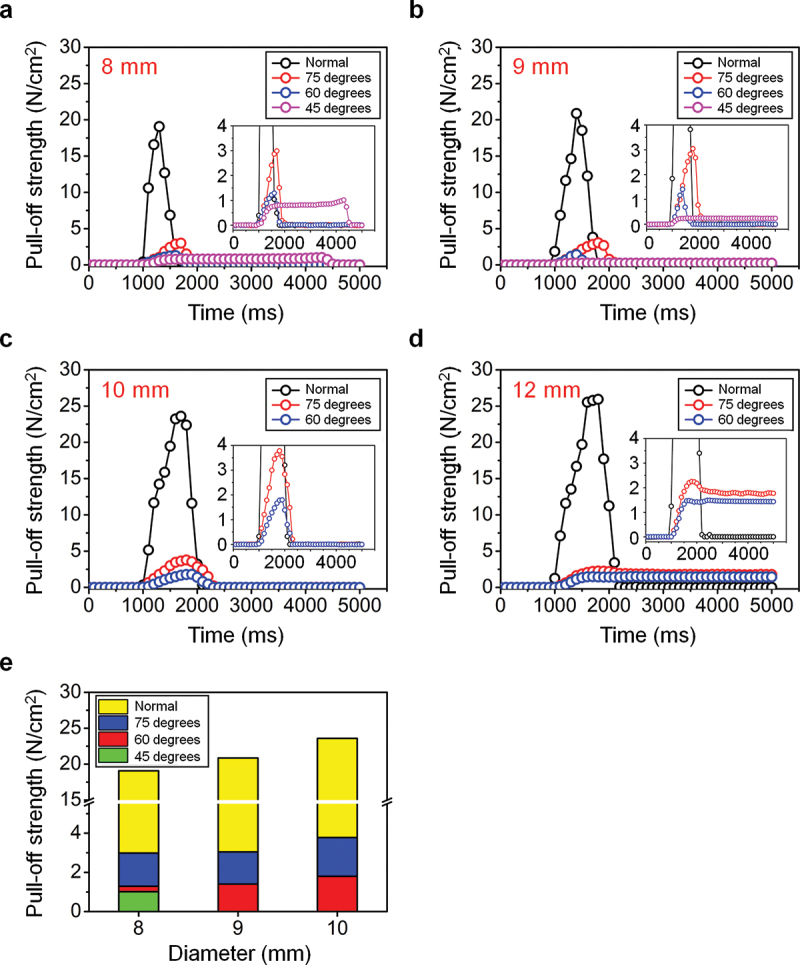
Pull-off strength measurements of (a) 8, (b) 9, (c) 10, and (d) 12 mm-thick dry adhesive specimens versus time. Insets correspond to the magnified graphs at the same time domain. (e) Maximum pull-off strength of dry adhesives with different diameters upon detachment condition.

**Figure 4.**
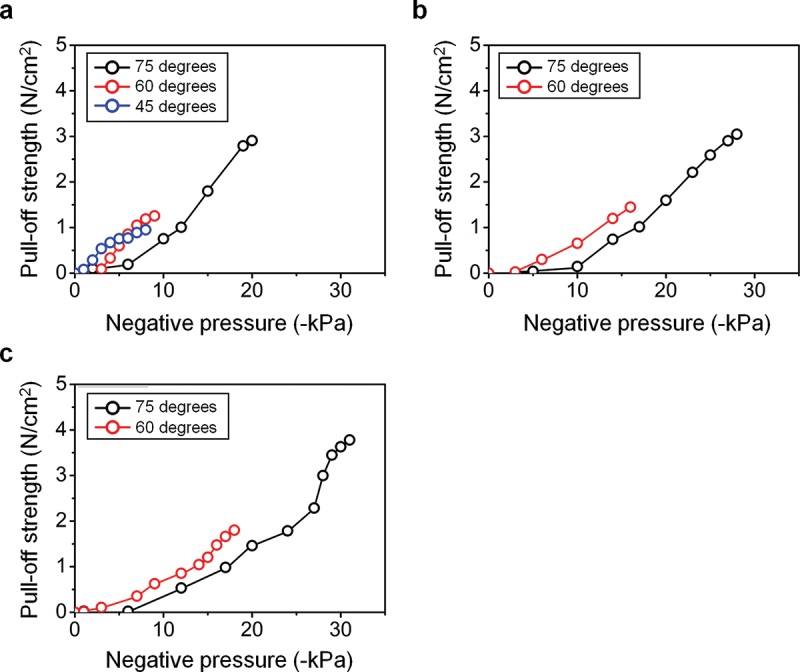
Graph of the pull-off strength versus applied negative air pressure until detachment of (a) 8, (b) 9, and (c) 10 mm-thick specimens.

The results of the pull-off strength derived from the detachment experiments of the dry adhesive are shown based on its diameter and on the vacuum pressure applied on the models in Figure 3. The graphs exhibit the curve between time and measured pull-off strength of each actuator model based on the different diameters of the dry adhesives (8, 9, 10, and 12 mm). When the dry adhesives are lifted by the robot arm following a normal direction without air actuation, the pull-off strength gradually increases from zero to maximum value during the detachment process and returns to zero after the process is completed. The maximum pull-off strength was measured as ~20 N cm^−2^ for the dry adhesive with a diameter of 8 mm and slightly increased by up to ~21, 23, and 26 N cm^−2^ as the diameter of the dry adhesive increases by 9, 10, and 12 mm, respectively. In cases of successful detachment by air actuations, the pull-off strength gradually increases to the maximum value and decreases after passing the apex. As the diameter increases, the maximum pull-off strength of the actuator models θ = 60° and 75° increases from 1.6 to 2.0 N cm^−2^ and from 3.0 to 3.8 N cm^−2^, respectively, representing approximately 8% and 15% of the normal detachment for each. Meanwhile, the pull-off strength increases to a certain extent and remains constant when the detachment fails. The actuator model of 45° manages to detach the dry adhesive of diameter 8 mm, whereas the 9 mm specimen is not taken off (Figure 3(a,b)). Similarly, the 60° and 75° models can detach the 10 mm sample but not the one of 12 mm (Figure 3(c,d)). As shown in Figure 3(e), a relatively low pull-off strength is measured in each case of actuation compared to the detachment without actuation. This is a remarkable result, which demonstrates that easy detachment could be achieved by operating the soft actuator. Figure 4 shows the detailed relations between pull-off strength and vacuum pressure applied to the actuators in the cases of the conformal detachment of the dry adhesive samples upon their diameters. The pull-off strength increases steadily until the detachment as the absolute value of the applied air pressure increases (Figures 4(a,c)). Moreover, for the same actuator model, lower air pressure is required to detach the dry adhesive as its diameter increases. Although both 60° and 75° models can detach the 8, 9, and 10 mm samples, a higher value of the applied air pressure is required for the 75° model to detach the dry adhesive compared to the 60° model. In detail, the 75° model successfully detached the 10 mm sample at the pressure of 31 kPa, while the 60° model demanded 18 kPa.

### 3.3. Analysis of the easy detachment

**Figure 5.**
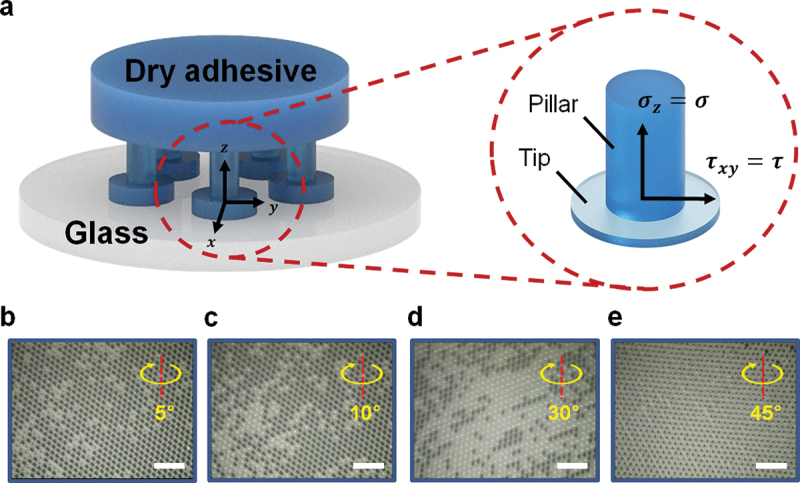
Schematic and images of micropillars in contact with a flat glass. (a) A Cartesian coordinate system and direction of stress applied to the micropillar. Images of the contact areas of the micropillars with the glass after clockwise rotation by angles of (b) 5°, (c) 10°, (d) 30°, and (e) 45°. The scale bar is 100 μm.

When vacuum pressure is applied to the soft actuators, the walls of oblique air chambers collapse inward and lead to tension. The vertical and horizontal components of the tensions cause the actuators to contract and twist, thus providing the interface with coupled normal stress and shear stress, respectively [35,44]. As shown in Figure 5(a), in two dimensions, a second-order stress tensor at the center of a micropillar can be written as follows:

(2) $$\sigma =\left[\begin{array}{cc} \sigma_{z} & \tau_{xy}\\ \tau_{xy} & 0 \end{array}\right]=\left[\begin{array}{cc} \sigma & \tau \\ \tau & 0 \end{array}\right],$$

where $\sigma_{z}=\sigma$ is the normal stress in the vertical direction, and $\tau_{xy}=\tau$ is the shear stress horizontal to the center of a top of the micropillar applied by the coupled motions. For the detachment of each micropillar, the relationship between σ and τ at the moment of its detachment can be described by an equation of Mohr’s circle given by:

(3) $${\left(\frac{\sigma }{2}\right)}^{2}+\tau^{2}=\;R^{2},$$

where *R* is the value of maximum in-plane shear stress for detachment. When a pillar is detached in the vertical direction without shear stress, Equation (3) can be modified as follows:

(4) $${\left(\frac{\sigma_{0}}{2}\right)}^{2}=\;R^{2},$$

where $\sigma_{0}$ is pull-off strength of each micropillar. By substituting Equation (4) into (Equation 3), the relationship between the σ and τ, which is required for the detachment and the pull-off strength, can be described as follows:

(5) $${\left(\frac{\sigma }{2}\right)}^{2}+\tau^{2}=\;{\left(\frac{\sigma_{0}}{2}\right)}^{2}.$$

Therefore, the micropillar can be detached when the complex stresses increase and reach a value exceeding $\sigma_{0}$. Figure 5(a) shows the schematic of the interface between microtips and a glass after the application of different twisting angles. In elastic region, shear stress can be expressed by,

(6) τ=Gγ

where G is shear modulus, and γ is shear strain.

From equation 6, shear stress is proportional to the value of shear strain. Therefore, the twisting motion results in an increase in shear stress, which leads to a reduction in pull-off strength. Also, due to the increase in shear stress, crack propagation was initiated (Figure 5(b–d)). The crack propagates as the motions continue, thus decreasing the pull-off strength of dry adhesive, which is proportional to the number of micropillars in contact with the glass [21,45]. As a result, the micropillars end up with elastic bending (Figure 5(e)), and the facile detachment can be achieved by low pull-off strength.

To detach the dry adhesive with the low pull-off strength, shear stress applied to the dry adhesive must exceed the threshold region at which the bonds rupture and detachment occurs [15,19]. As the value of the chamber angle *θ* increases, the cross-section of the air chambers increases, and it may result in the increased torsional stiffness of the soft actuator [35]. Thus, a relatively high magnitude of negative pneumatic pressure is required for the model of angle 75° to exceed the region and high pull-off strength is generated when the dry adhesive is finally detached. In the case of the model of angle 45°, despite the reduced torsional stiffness, the 45° angle model exhibits lower controllable adhesion energy compared to the 60° and 75° angle models. When the width of the air chambers is normalized by the length of the side wall (c/e) depicted in Figure 2(b,c), the c/e of the 45° angle model is about twice as high as the others. Perhaps, this leads to the contact of the side walls having a low-magnitude vacuum pressure and tensions derived from the walls remaining constant [44,46]. As a result, the model can exert low stress upon the dry adhesive and exhibit low controllable adhesion energy.

## 4. Application

**Figure 6.**
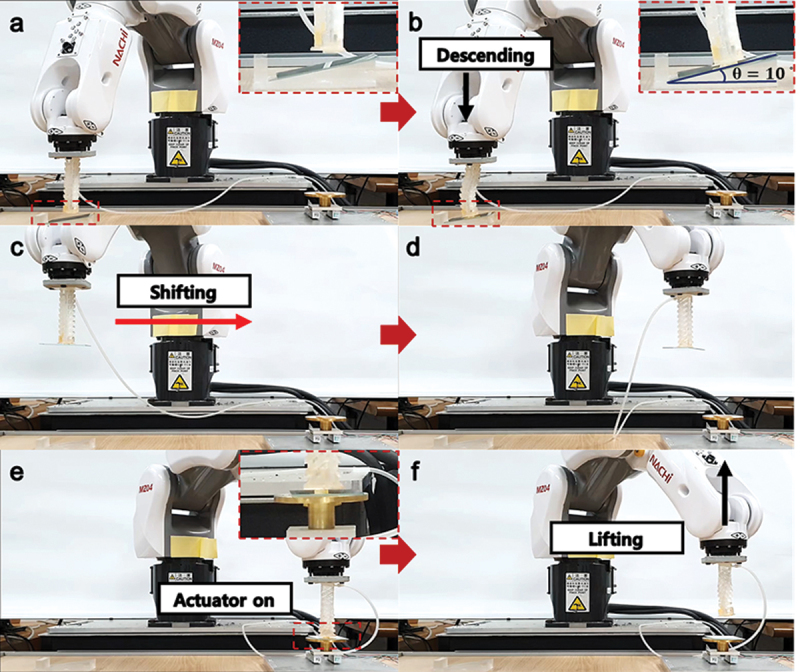
Demonstration of the glass transfer system assembled with a robot arm, dry adhesive, and the actuator model. (a) Alignment of the actuator model above a glass on a slope by a robot arm. (b) Attachment of the model on a glass while bent. (c) Lifting and (d) disposition of a glass on a particular site. (e) and (f) Detachment of the glass by the twisting of the actuator model.

Based on the above experiments, a soft actuator applied-glass transfer system with a 6-DOF robot arm was suggested as a feasible application as shown in Figure 6. In the time-lapse images, the actuator came into contact with the tilted glass and picked it up by adapting the 10° angle of the slope due to the flexibility of the soft actuator (Figures 6(a,b)). Then, the glass was lifted while attached to the dry adhesive and delivered to a particular site as the robot arm moved horizontally (Figures 6(c,d)). When the glass arrived on the site, it was anchored on the bottom, which was coated by a double-sided tape. The fix glass was detached from the dry adhesive as negative air pressure was applied to the actuator (Figures 6(e,f)). Through the system, the glass substrate was safely transferred without fall and fracture during the transfer.

## 5. Conclusion

In this study, we introduced a novel vacuum-powered soft actuator featuring a dry adhesive, which can safely transfer the glass substrate by coupled contraction and twisting. To make these actuators, design concepts containing a monolithic air channel, oblique air chambers, and back-to-back bonding were presented. The dry adhesive samples underneath the actuator models were attached to flat glass, and the pull-off strength generated by the actuation of the models was measured along with the applied air pressure.

We present a summary of our findings below:

1. The vacuum-powered soft actuator was introduced. Various prototypes were suggested, and the soft actuator that can be easily manipulated by the negative pneumatic pressure was achieved.
2. The facile detachment of the dry adhesive was confirmed. The experiment results revealed that a relatively low pull-off strength was generated compared to the generally used vertical detachment method of the artificial dry adhesive, which differed based on the chamber angles of the models.
3. The feasible application was illustrated. On the basis of (1) and (2), a novel glass transfer system was proposed with the 6-DOF robot arm, and its good performance was confirmed. This system can not only lift a tilted glass without rupture but also safely deliver the glass to the target area.

Through this study, the pneumatic powered soft actuator with the dry adhesive, which is capable of attachment with glass, is presented from the feasible application perspective view. We believe that this study can provide insights into the usage of the pneumatic powered soft actuators as end effectors for the active adhesion control of artificial dry adhesives.

## Supplementary Material

Supplemental Material

Supplemental Material
