# Supplementary material for: Vacuum-powered soft actuator with oblique air chambers for easy detachment of artificial dry adhesive by coupled contraction and twisting
Source: Sci Technol Adv Mater. 2023 Nov 9;24(1):2274818. doi: 10.1080/14686996.2023.2274818 (PMC10653703; doi:10.1080/14686996.2023.2274818)
Supplement: Supplemental Material [file TSTA_A_2274818_SM4311.docx]

Supporting Information

**Vacuum-powered soft actuator with oblique air chambers for easy detachment of artificial dry adhesive by coupled contraction and twisting**

Seung Hoon Yoo^1^, Minsu Kim^1^, Han Jun Park^1^, Ga In Lee^1^, Sung Ho Lee^2,*^, and Moon Kyu Kwak^1,*^

**Contents**

**Figure S1.** Schematic illustration for fabrication of the artificial dry adhesive. (a) SI mold with the mushroom-shaped structure. (b) PDMS poured on the mold. (c) Demolding of the cured dry adhesive.

**Figure S2.** Overall fabrication process of the actuator body. (a) Schematic illustration of the fabrication process. (i) Mold fabricated by the 3D printer. (ii) Combined mold parts on which degassed PDMS was poured. (iii) PDMS on the mold cured at 70 ℃ for 2 h. (iv) Demolded and (v) assembled halves of the model. (b) Completely combined actuator model.

**Figure S3.** Schematics for the pull-off strength measurements of the actuator models. (a) A preload given to the dry adhesive by a robot arm. (b) Detachment of the dry adhesive in the vertical direction. (c) Detachment of the dry adhesive by actuation of the actuator models. (d) Time lapse images of the pull-off strength measurements.

**
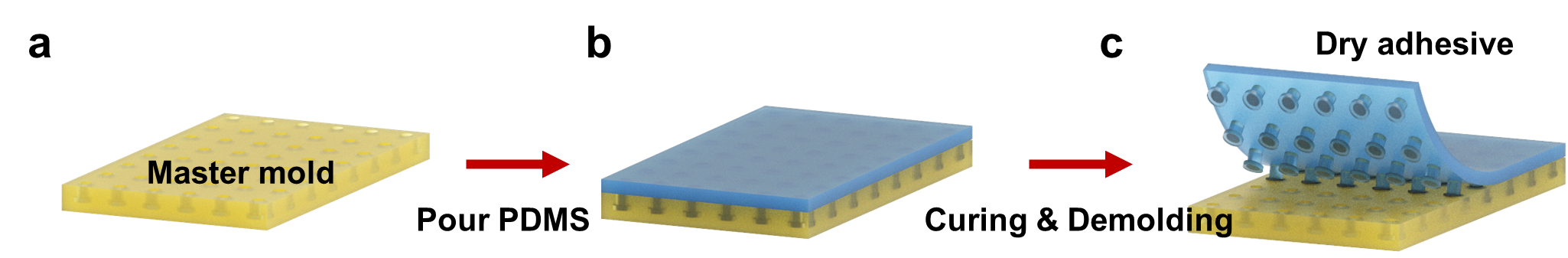
**

Figure S1. Schematic illustration for the fabrication of the artificial dry adhesive. (a) SI mold with the mushroom-shaped structure. (b) PDMS poured on the mold. (c) Demolding of the cured dry adhesive.


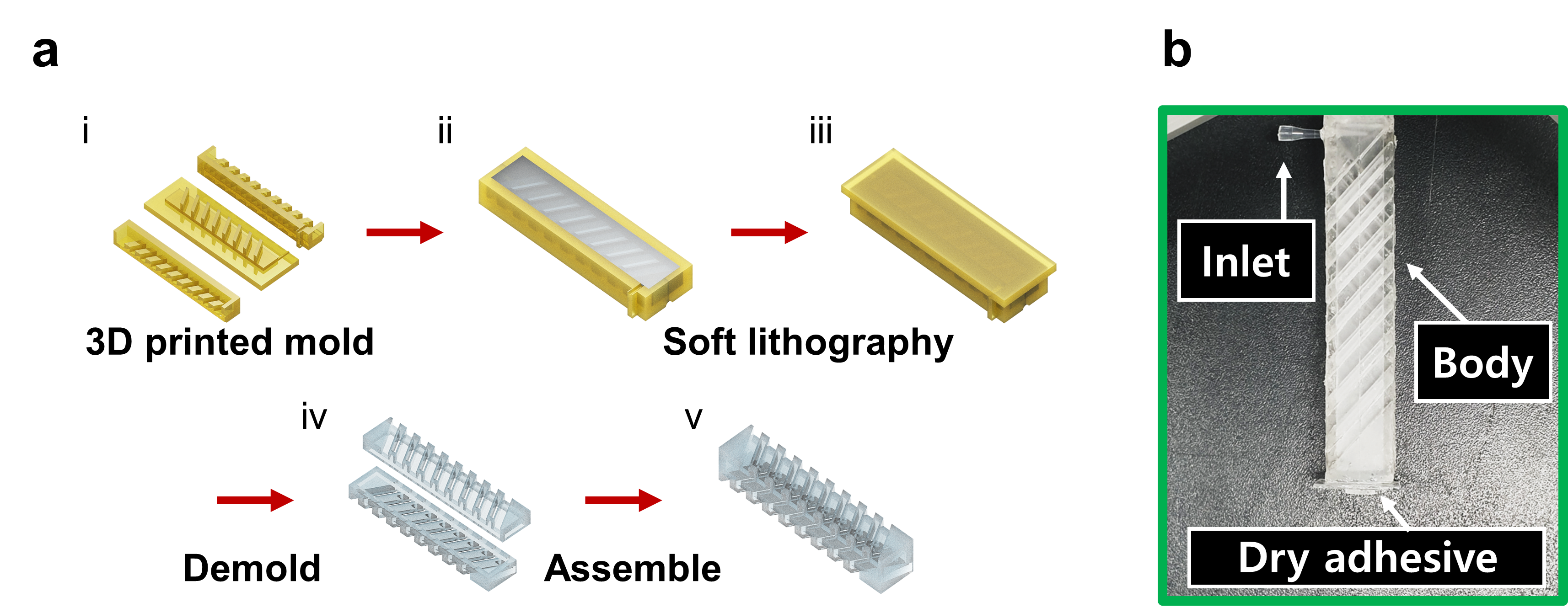


Figure S2. Overall fabrication process of the actuator body. (a) Schematic illustration of fabrication process. (i) Mold fabricated by the 3D printer. (ii) Combined mold parts on which degassed PDMS was poured. (iii) PDMS on the mold cured at 70 ℃ for 2 h. (iv) Demolded and (v) assembled halves of the model. (b) The completely combined actuator model.


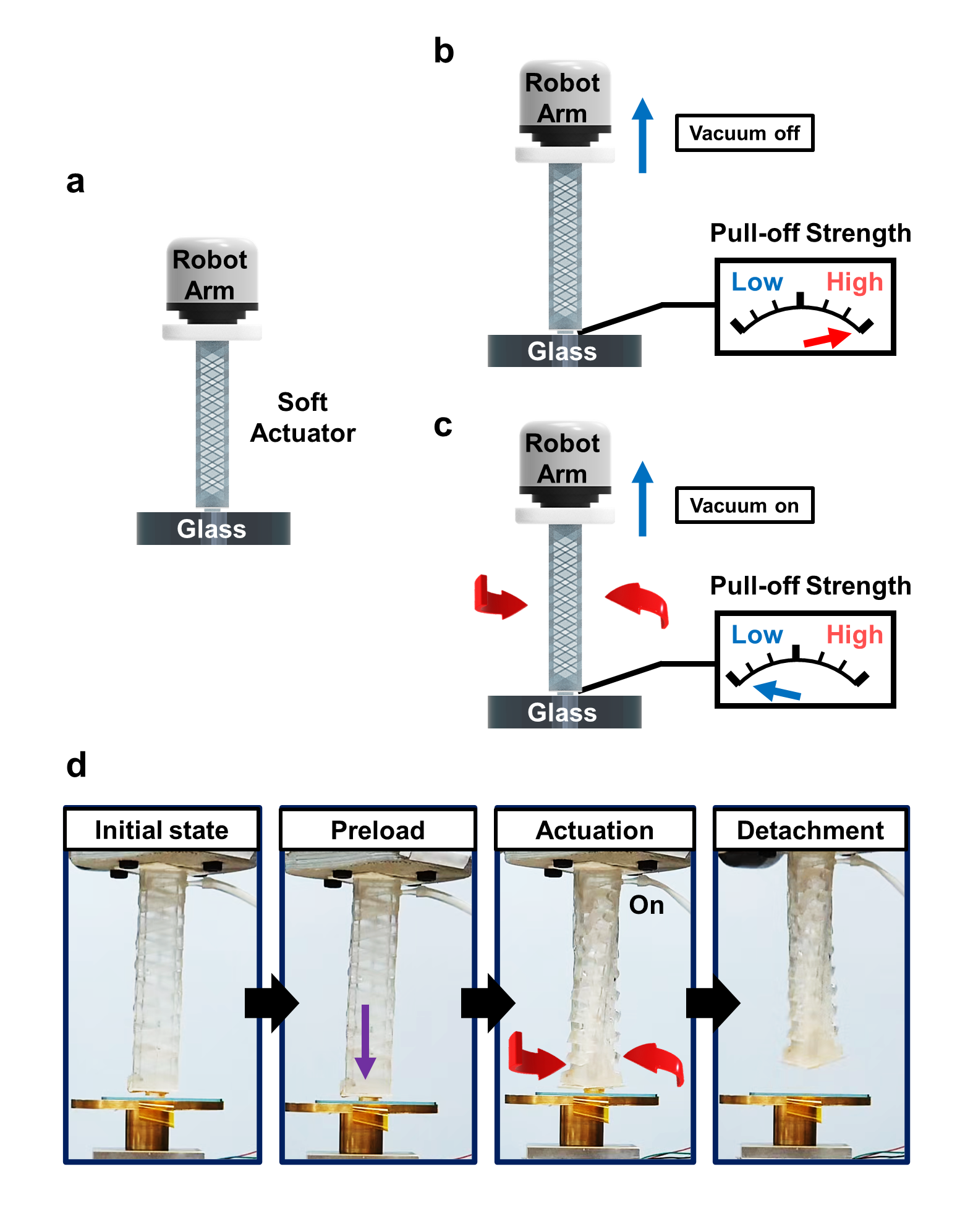


Figure S3. Schematics for the pull-off strength measurements of the actuator models. (a) A preload given to the dry adhesive by a robot arm. (b) Detachment of the dry adhesive in vertical direction. (c) Detachment of the dry adhesive by actuation of the actuator models. (d) Time lapse images of the pull-off strength measurements.
